# Delayed Bladder Perforation Resulting From Large Bladder Stone and Gluteal Abscess Caused by Pelvic Plate Loosening: A Case Report

**DOI:** 10.7759/cureus.61543

**Published:** 2024-06-02

**Authors:** Shun-An Kan, Ya-Che Lee, Hui-Chu Tsai, Ying-Ying Su, Fang-Chieh Lien

**Affiliations:** 1 Medicine, National Yang Ming Chiao Tung University, Taipei, TWN; 2 Urology, Ditmanson Medical Foundation Chia-Yi Christian Hospital, Chiayi, TWN; 3 Radiology, Ditmanson Medical Foundation Chia-Yi Christian Hospital, Chiayi, TWN; 4 Orthopedics, Ditmanson Medical Foundation Chia-Yi Christian Hospital, Chiayi, TWN

**Keywords:** bridge plate, gluteal abscess, plate loosening, delayed bladder perforation, pelvic ring injury

## Abstract

Delayed bladder injuries resulting from screw or plate loosening, following pelvic ring fractures are rare, and this complication could be prevented. A 63-year-old woman presented with dysuria and lower abdominal pain, 13 years after the open fixation of a pelvic injury. Computed tomography revealed a 5-cm bladder stone and two migrated screws. Six months after the stone was removed, an abscess was noted over the left gluteal region. During the removal of the screw and abscess debridement, we accidentally observed that the anterior pelvic bridge plate had eroded into the bladder and had multiple bladder stones attached. After the involved hardware was removed, the abscess was debrided and the bladder was repaired. The patient did not have further urinary tract infections or urinary symptoms. In patients with pelvic ring fractures, we recommend placing the bridge plate on the superior side of the pubic symphysis to reduce the risk of bladder perforation in the event of plate or screw loosening. When a patient with a history of pelvic fixation presents with symptoms such as urinary tract infections, bladder stones, or even an abscess around the gluteal region, possible bladder perforation caused by the loosening of plates or screws should be considered.

## Introduction

Pelvic ring injuries are usually caused by high-energy traumatic collisions, such as traffic accidents, with 5%-6% of patients presenting with bladder injuries [1,2]. Among the treatment options for such injuries, open reduction and internal fixation is often performed in patients with unstable pelvic fractures [3]. However, delayed bladder injuries resulting from screw or plate loosening have only been reported in a few studies [4-7]. Most of the patients in these studies presented with symptoms of recurrent urinary tract infections (UTIs), dysuria, or spontaneous voiding of a screw. In this paper, we report a case of delayed bladder perforation resulting from a large bladder stone and an abscess over the gluteal region caused by screw loosening.

## Case presentation

**Figure 1:**
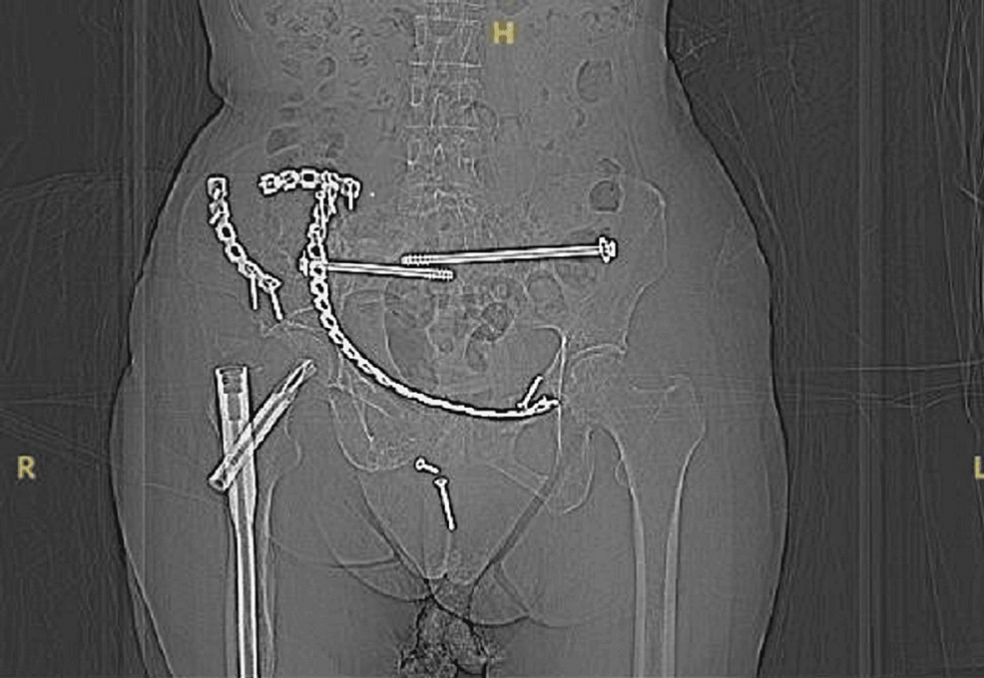
Radiograph upon admission showing the anterior pelvic bridge plate extending from the right ilium to the left pubic region, two reconstruction plates over the right iliac fossa, and three sacral screws inserted to fix bilateral sacral fractures. Three migrated screws were detected in the pelvic region.

**Figure 2:**
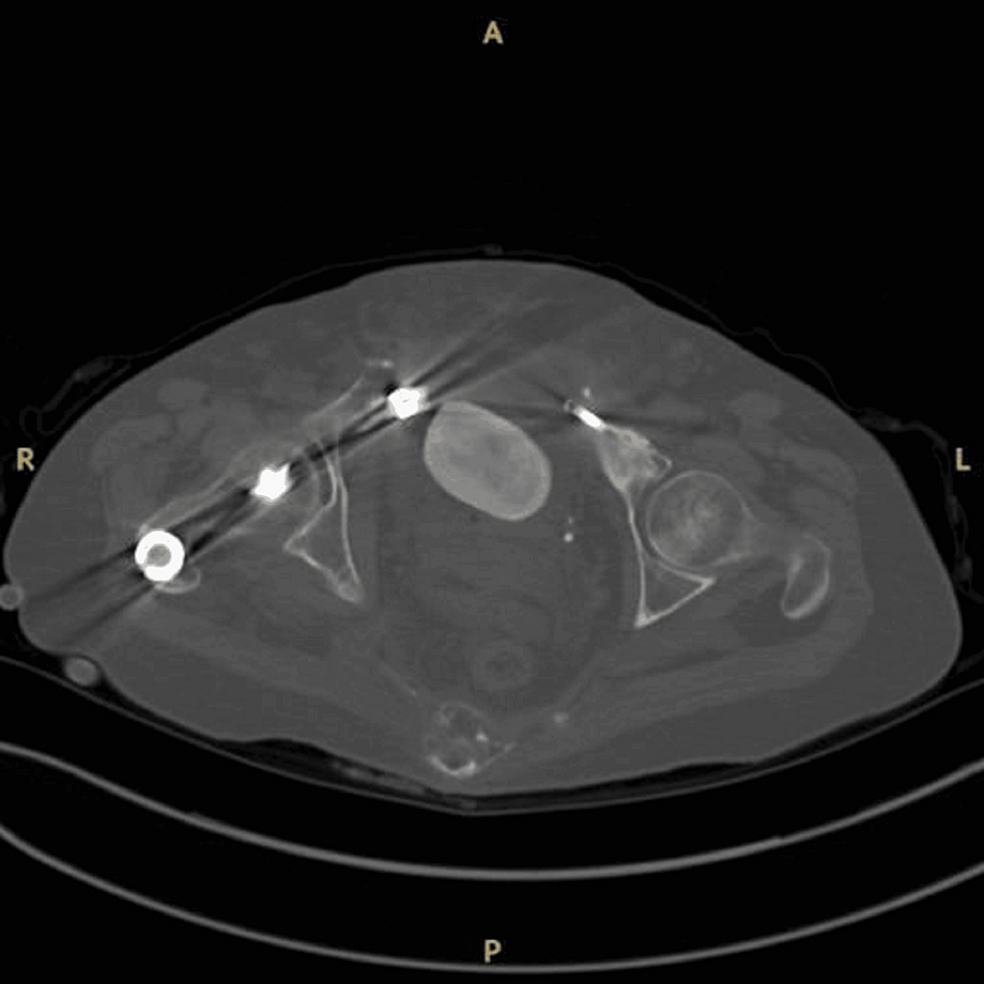
Abdominal computed tomography showing a 5-6 cm bladder stone.

A 63-year-old woman was admitted to our emergency department with chief concerns of lower abdominal pain when defecation and voiding for years. Approximately 13 years prior to her presentation, the patient had a traffic accident, which resulted in multiple fractures of the bilateral iliac bone, sacrum, bilateral superior pubic ramus, and right femur. She was then subjected to open reduction and internal fixation in a community hospital; this procedure involved the use of an anterior pelvic bridge plate extending from the right ilium to the left pubic region and the placement of two reconstruction plates over the right iliac fossa. Three sacral screws were also inserted to fix bilateral sacral fractures (Figure 1). Thirteen years after the surgical procedure, the patient visited our emergency department with lower abdominal pain. Bedside sonography revealed a distended bladder containing more than 600 mL of urine and bilateral hydronephrosis. Noncontrast abdominal computed tomography (CT) revealed a large bladder stone (5-6 cm; Figure 2). A blood test showed leukocytosis with an elevated C-reactive protein level (11.801 mg/dL), indicative of a urinary tract infection (UTI). Therefore, the patient underwent transurethral cystolithotripsy and received antibiotics. She was discharged in stable condition.

**Figure 3:**
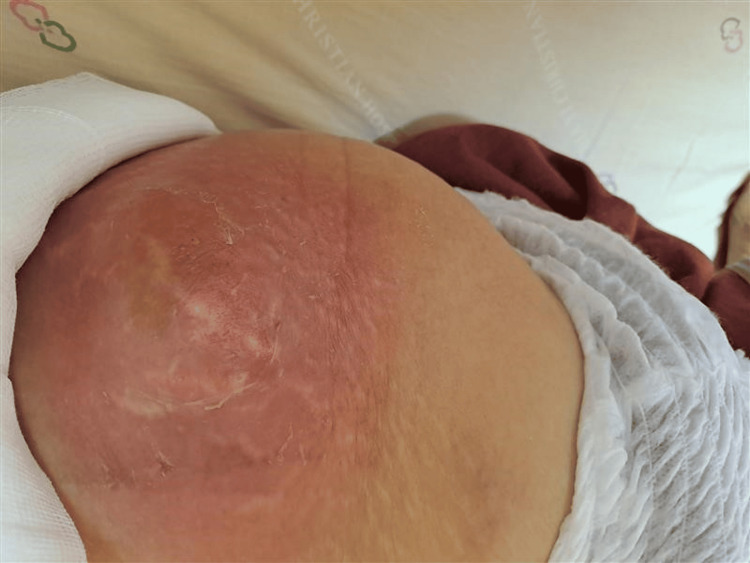
The abscess over the left gluteal region.

**Figure 4:**
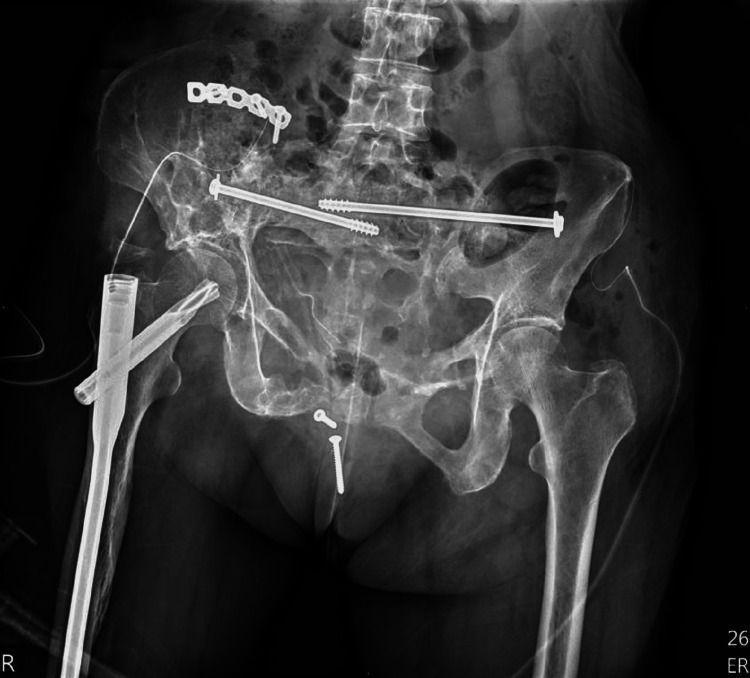
The loosening plate extending from the right ilium to the left pubic region and one reconstruction plate were removed.

Six months later, the patient noticed local swelling with redness in the left gluteal region, which enlarged progressively. Moreover, her lower abdominal pain persisted. She thus visited our outpatient department and underwent aspiration for the left hip mass (Figure 3). However, her symptoms persisted, and she again visited our orthopedic outpatient department. Laboratory tests revealed elevated C-reactive protein levels (7.055 mg/dL) and leukocytosis resulting from a left gluteal abscess. The patient requested the removal of the three migrated screws around her pelvic area; accordingly, she was admitted to the orthopedic ward for surgical screw removal and abscess debridement. However, during the surgery, a 5-cm bladder rupture was noted. Moreover, one segment of the bridge plate was detected in the bladder cavity and was covering the stone. The bridge plate had multiple bladder stones attached and was removed. Furthermore, one reconstruction plate and two of the three cannulated screws were removed sequentially (Figure 4). The bladder wall defect was debrided and closed using two layers of 3-0 catgut and Vicryl sutures. The abscess over the left gluteal region was also debrided. Subsequent wound culture revealed the presence of oxacillin-resistant *Staphylococcus aureus*. The patient underwent another debridement of the abscess 10 days later because of poor healing.

**Figure 5:**
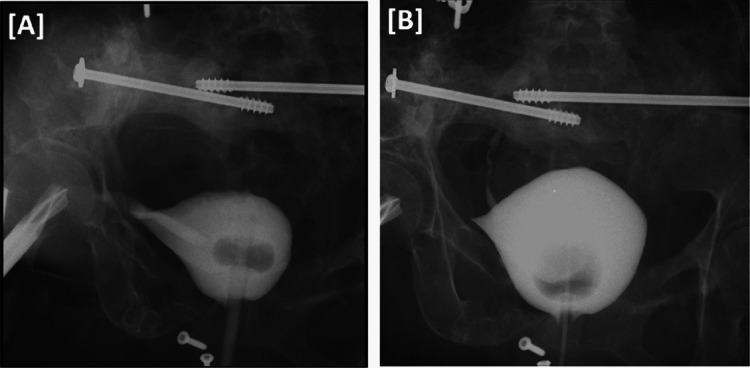
Cystogram performed. (A) Cystogram performed two weeks after hardware removal and bladder repair showing the tract formed by the bridge plate from the bladder to the retroperitoneal space. (B) Cystogram performed one month after surgery showing a decrease in the tract with no significant leakage.

After the surgery and 14 days of antibiotic treatment, the patient was discharged in stable condition. During two-month follow-up, the abscess region recovered almost completely. Moreover, the patient’s lower abdominal pain and dysuria were resolved. Follow-up cystograms revealed good healing of the bladder rupture (Figures 5A, 5B).

## Discussion

UTIs and bladder stones are both common and could be caused by foreign bodies. To the best of our knowledge, bladder perforation and UTIs resulting from the intravesical migration of a pelvic plate are uncommon. Moreover, large bladder stones and gluteal abscesses resulting from a pelvic plate have not been reported previously.

Fridman et al. reported the case of a patient who presented with symptoms of dysuria, acute hematuria, and spontaneous voiding of a screw seven years after fixation [4]. Heetveld et al. also reported the case of a patient with recurrent UTIs caused by a spontaneously voided screw, which was used for symphyseal plate fixation nine years earlier [5]; they later detected a fistula from the bladder to the defect caused by the screw and hypothesized it to be the cause of the recurrent UTIs. In the case presented by Babu et al., the patient experienced recurrent UTIs 11 years after the open fixation of a pelvic injury [6]. Cystoscopy revealed that the pelvic plate had eroded into the bladder, causing recurrent infections; this observation is similar to that noted in our case. The patient had no further UTI after the involved plate was removed, although two screws remained in the pelvis after pelvic plate removal. Because the retained screws were in the soft tissue and far from the urinary tract system, similar problems are unlikely to occur in the future. During the follow-up period, the patient did not report any symptoms or signs related to these retained screws. Shon et al. also reported the case of a patient with plate breakage and screw perforation into the bladder, resulting in hematuria and dysuria [7].

Our case differs from previously reported cases, in which patients mostly presented with dysuria, hematuria, or recurrent UTIs. Some cases involved obvious signs of hardware failure, such as a spontaneously voided screw or a broken plate [4,6,7]. Our case involved a UTI accompanied by a large bladder stone measuring 5-6 cm. An abscess over the left gluteal region was observed months after stone removal. We hypothesized that the loosening of the screw caused the plate to protrude into the bladder. As a foreign body, the plate resulted in the formation of the bladder stone and the development of the UTI. Moreover, the plate possibly formed a tunnel from the bladder to the left gluteal area, resulting in infection and abscess formation in the left gluteal region.

## Conclusions

Our case exhibited a rare complication of delayed bladder perforation 13 years after pelvic ring fixation surgery using a bridge plate. The bridge plate was placed on the posterior side of the pubic symphysis, which was right next to the bladder. Consequently, the patient had a relatively high risk of bladder perforation in the event of plate or screws loosening. Hence, in patients with pelvic ring fractures, we recommend placing the bridge plate on the superior side of the pubic symphysis to reduce the complication rate. Furthermore, if a patient with a history of pelvic fixation presents with symptoms of a UTI, bladder stones, or even an abscess around the gluteal region, possible bladder perforation caused by the loosening of plate or screws should be considered, and cystoscopy or CT should be performed to assess the stability of the hardware.
